# Differential effects of hypoxia on motility using various in vitro models of lung adenocarcinoma

**DOI:** 10.1038/s41598-024-70769-w

**Published:** 2024-09-03

**Authors:** Sára Eszter Surguta, Marcell Baranyi, Laura Svajda, Mihály Cserepes, Ivan Ranđelović, Enikő Tátrai, Balázs Hegedűs, József Tóvári

**Affiliations:** 1 Department of Experimental Pharmacology and the National Tumor Biology Laboratory, National Institute of Oncology, Budapest, 1122 Hungary; 2 School of Ph.D. Studies, Semmelweis University, Budapest, 1085 Hungary; 3 Department of Pathology, Forensic and Insurance Medicine, Semmelweis University, Budapest, 1091 Hungary; 4 Department of Thoracic Surgery, University Medicine Essen - Ruhrlandklinik, University Duisburg-Essen, 45239 Essen, Germany

**Keywords:** Cancer, Lung cancer, Metastasis

## Abstract

Lung cancer is the leading cause of cancer-related death globally. Metastasis is the most common reason of mortality in which hypoxia is suggested to have a pivotal role. However, the effect of hypoxia on the metastatic potential and migratory activity of cancer cells is largely unexplored and warrants detailed scientific investigations. Accordingly, we analyzed changes on cell proliferation and migratory activity both in single-cell migration and invasion under normoxic and hypoxic conditions in lung adenocarcinoma cell lines. Alterations in crucial genes and proteins associated with cellular response to hypoxia, epithelial-mesenchymal transition, proliferation and apoptosis were also analyzed. Generally, we observed no change in proliferation upon hypoxic conditions and no detectable induction of apoptosis. Interestingly, we observed that single-cell motility was generally reduced while invasion under confluent conditions using scratch assay was enhanced by hypoxia in most of the cell lines. Furthermore, we detected changes in the expression of EMT markers that are consistent with enhanced motility and metastasis-promoting effect of hypoxia. In summary, our study indicated cell line-, time of exposure- and migrational type-dependent effects of hypoxia in cellular proliferation, motility and gene expression. Our results contribute to better understanding and tackling cancer metastasis.

## Introduction

Lung cancer is the most common cancer type and the leading cause of death related to cancer worldwide^1^. Furthermore, despite the advances in lung cancer therapies, 5 year survival rate remains low at 5–15%^2^. Poor prognosis is largely due to the lack of obvious symptoms in the early stage and consequent late diagnosis of the advanced, metastatic disease^3^. The most common reason of disease progression and mortality for lung cancer patients is metastasis^4^. Metastasis is a complex process upon which the malignant cells acquire the ability to migrate from the primary tumor, invade surrounding tissues, enter the circulatory system, and form secondary tumors in distant organs. Studies focusing on tumor progression and metastasis suggest that hypoxia may be an important contributing factor to this process^5^.

Therefore, investigation of the molecular mechanisms behind the development of lung cancer under hypoxia is crucial to establish more rational therapeutic approaches for the treatment of lung adenocarcinoma (LUAD) patients.

Hypoxia (oxygen deprivation) is a prevalent occurrence in the majority of malignant solid tumors. Several factors influence the oxygen levels in tumors such as tumor size, stage, the heterogeneity of the tumor and the initial oxygenation of the tissue^6,7^. For example, the median oxygen level of the lung in physoxia is 5.6% O_2_ whereas the hypoxic tumor tissue of lung cancer patients is between 1.9 and 2.2% O_2_^8^.

Generally, three types of tumor hypoxia can be distinguished in the tumor microenvironment based on its duration: acute, chronic, and intermittent hypoxia. Although there is a lack of consent in the exact definitions, acute hypoxia can be defined by short-term, less than 24 h of exposure to an environment with 1% O_2_ or less^9^. On the other hand, chronic hypoxia is defined by long exposure for more than 48 h to low oxygen levels. Both acute and chronic hypoxia can be observed in an intermittent phase by reoxygenation of hypoxic tissue and hypoxic-oxic cycles^10^.

Regarding hypoxia-induced molecular changes, a family of transcriptional regulators known as hypoxia-inducible factors (HIFs) were identified as the primary mediators of cellular response to hypoxia three decades ago^11^. It was found that under hypoxia, HIF-1α accumulate in response to decreased cellular oxygen levels. Conversely, in normoxia they undergo degradation via proteasome^12^. All three HIF family members—HIF-1, HIF-2, and HIF-3 -^13^ have the ability to bind to hypoxia-response elements (HREs). This binding activates distinct transcriptional responses, contributing to the regulation of hypoxia signaling and maintaining oxygen homeostasis within cells^14,15^. One major metastasis-promoting process that can be modulated by hypoxia is the epithelial-mesenchymal transition (EMT). In general, during EMT, cells lose the expression of certain epithelial markers such as E-cadherin, while increasing the expression of mesenchymal markers like N-cadherin or vimentin^16,17^. Cells that undergo epithelial-mesenchymal transition gain mesenchymal-like behavior, facilitating their migration away from the primary tumor site through various migration strategies^18^, including multicellular, collective, and single-cell mesenchymal migration^19^.

Numerous studies aimed to investigate the effects of hypoxia on cell motility in vitro; however, these findings are often conflicting and utilized varying experimental setups^20^. Thus, further studies are urgently needed for better understanding of this crucial process in tumor progression.

In this study, our aim was to examine the impact of chronic hypoxia on expression of EMT markers, proliferation and motility in a panel of human lung adenocarcinoma cell lines. We found that chronic hypoxia may act as an acute stressor for the cells based on reduced expression of proliferation markers. We also demonstrated that changes in the expression of EMT markers in several cell lines are consistent with the metastasis-promoting effect of hypoxia. Also, we demonstrated that vimentin shows EMT-related morphology under hypoxic conditions in lung adenocarcinoma cells. Furthermore, we found differential motility responses that depended on the cellular density. More specifically, we found a general decrease of motility upon analysis of single-cell movement, while hypoxia enhanced migratory activity under confluent conditions. In conclusion, our study provides an extensive analysis of the differential effects of hypoxic conditions on lung cancer cell viability and migratory potential and warrants further studies to understand its role in shaping the tumor progression.

## Results

### The effect of hypoxia and CoCl_2_ on apoptosis and proliferation

In this study, we selected a panel of lung adenocarcinoma cell lines harboring major mutations commonly found in LUAD tumors, more specifically mutations of EGFR (H1975), KRAS (PF139) and BRAF (PF901) oncoproteins, furthermore, we also included one cell line with no known driver mutations (H838).

**Fig. 1:**
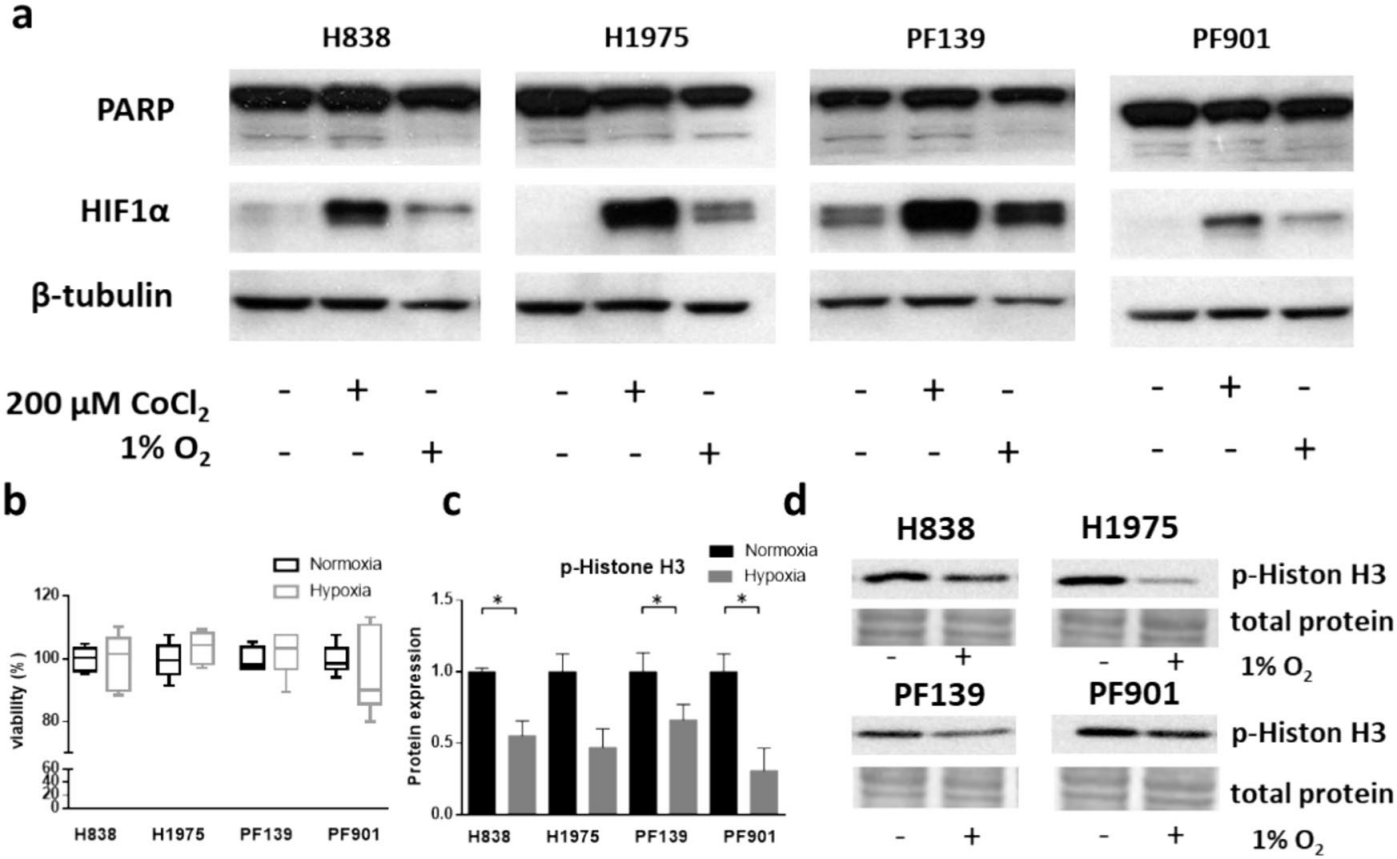
Effect of hypoxia and CoCl_2_ treatment on hypoxia, proliferation, and apoptosis-related protein expression. Cells were exposed for 48 h to normoxic (21% O_2_ control) or hypoxic (1% O_2_) conditions or 200 µM CoCl_2_ treatment (under 21% O_2_). (**a**) Representative Western blot images showing protein level changes upon different conditions. (**b**) Graph show the proliferation data obtained from SRB viability assay. (**c**) Graph shows the densitometric evaluation of investigated-Histone H3 protein, normalized to total protein and expressed relative to control (normoxic sample). (**d**) Representative Western blot images showing p-Histone H3 protein level changes upon hypoxic conditions. Data derived from three independent experiments plotted as mean ± SD. Asterisks show statistically significant differences. Statistical significance was established using unpaired t-test with Welch correction at p < 0.05. Uncropped blots can be found at supplementary information file.

To examine main cellular responses to hypoxia, we performed immunoblot analysis of changes in HIF-1α, proliferation marker p-Histone H3 and apoptosis based on the presence of cleaved PARP in in vitro cultured LUAD cells that were exposed to CoCl_2_ treatment or hypoxic conditions (1% O_2_) **(**Fig. 1**)**. As a generic signaling hub induced by hypoxia, HIF-1α was upregulated in all cell lines upon exposure to hypoxia or CoCl_2_ (Fig. 1a). Notably, CoCl_2_ is a hypoxia-mimetic agent, that blocks physiological HIF-1α protein degradation leading to its stabilization and accumulation. Regarding cell proliferation, we did not detect any significant changes based on SRB viability tests (Fig. 1b), however, we observed reduced p-Histone H3 protein expression under hypoxia (Fig. 1c,d). Neither condition induced apoptosis in any of the cell lines based on the lack of detectable cleaved PARP signal.

### Effects of hypoxia and CoCl_2_ on single-cell migration ability

**Fig. 2:**
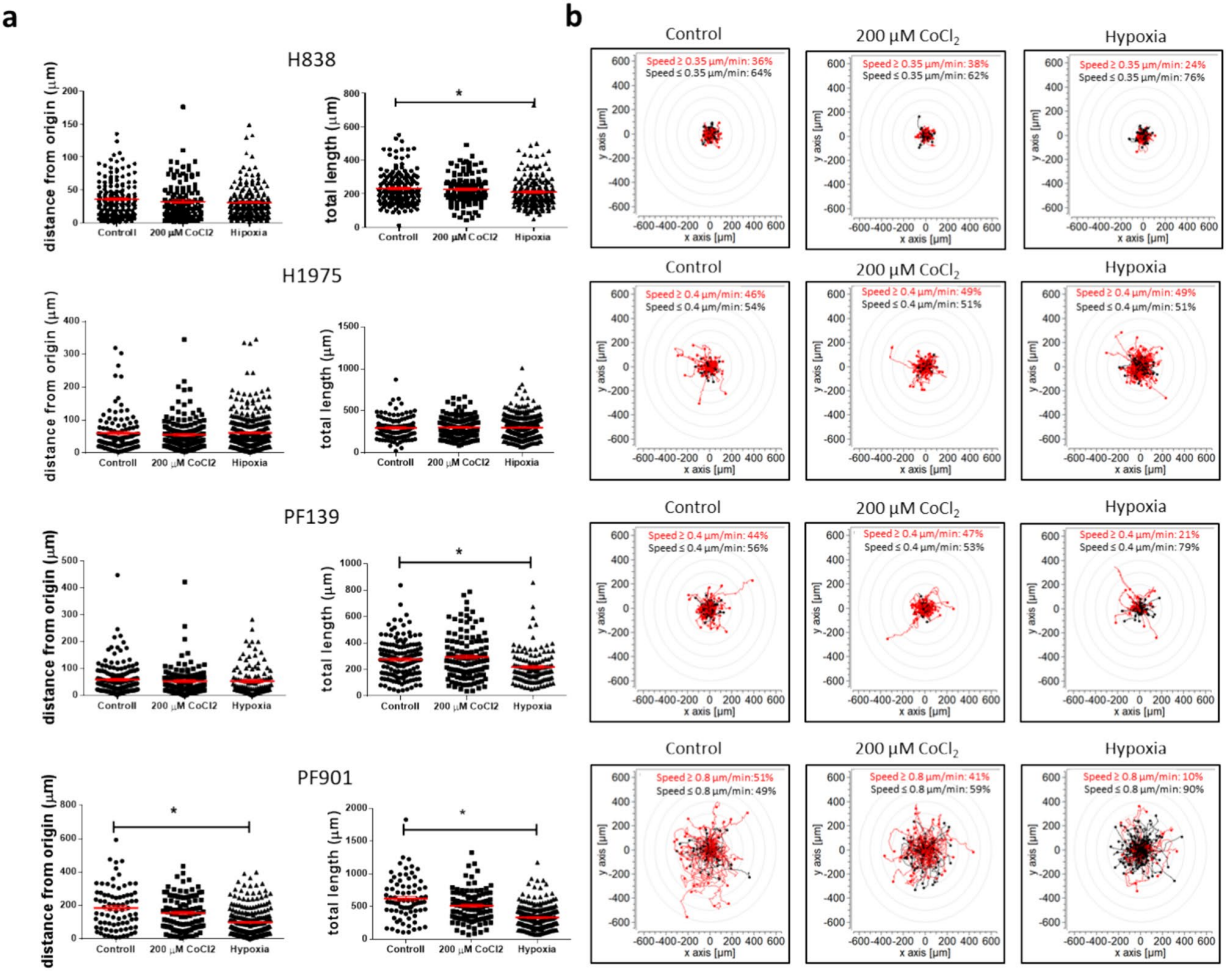
Effects of hypoxia on single-cell motility. (**a**) Motility of individual cells in the last 12 h frame of the 48 h-long exposure to normoxia (21% O_2_), 200 µM CoCl_2_, and hypoxia (1% O_2_). Only cells that were presented on the video for the whole time period were included in the analysis. The distance from the origin and the total length are plotted. (**b**) Images shows trajectories of the corresponding cells shown in the left panel (**a**). Trajectories that show higher speed than a cut-off value (showed in each image, adjusted to the speed of the given cell line) are colored red. Note the differences in baseline motility of the distinct cell lines. Asterisks mark statistically significant differences at p < 0.05. Data is derived from three independent experiments. Two independent wells were monitored for each experiment for normoxia and 200 µM CoCl_2_ treatment, while four independent wells were monitored for hypoxia. Normality was tested with the Shapiro-Wilk test. If the normal distribution was confirmed, one-way ANOVA with Tukey’s Multiple Comparison test was applied, otherwise Kruskal-Wallis test was used followed by Dunn’s multiple comparison post-test. Data plotted as mean ± SEM.

To characterize the effects of hypoxia on single-cell migratory activity of the four tumor cell lines, we performed time-lapse videomicroscopy. Tumor cells were allowed to migrate for 48 h under normoxic (21%O_2_), 200 µM CoCl_2_ (21%O_2_)_,_ and hypoxic (1%O_2_) conditions. Notably, pronounced differences could be observed in the migratory capacities of the four cell lines (Fig. 2a**).** Generally, CoCl_2_ treatment had no effect on cellular motility in any of the cell lines. However, in the case of hypoxic conditions, diverse, cell line-dependent changes could be observed.

In case of H838, which is the least motile cell line in this panel, there was an initial significant increase in motility in the first 24 h compared to the control, however, migratory activity dropped in the last 12 h of the experiment (Supplementary Fig. 1). No differences were seen in H1975 cell line between distinct conditions. Hypoxia significantly reduced single-cell migration of PF139 and PF901 cell lines throughout the experiment (Fig. 2**, **Supplementary Fig. 1, 3–4). Regarding PF139, it can be observed that there was a decrease in both distance from the origin and total length from the starting point (Supplementary Fig. 3). However, in the last 12 h, a significant difference was only observed in the total length under hypoxic conditions as compared to the control. PF901, the fastest cell line, showed a reduction in hypoxia over the entire study period in terms of total length (Supplementary Fig. 4). Moreover, in the last 12 h, both measured motility parameters were significantly reduced under hypoxic conditions (Fig. 2).

### Differential effect of hypoxia on invasion under confluent conditions

**Fig. 3:**
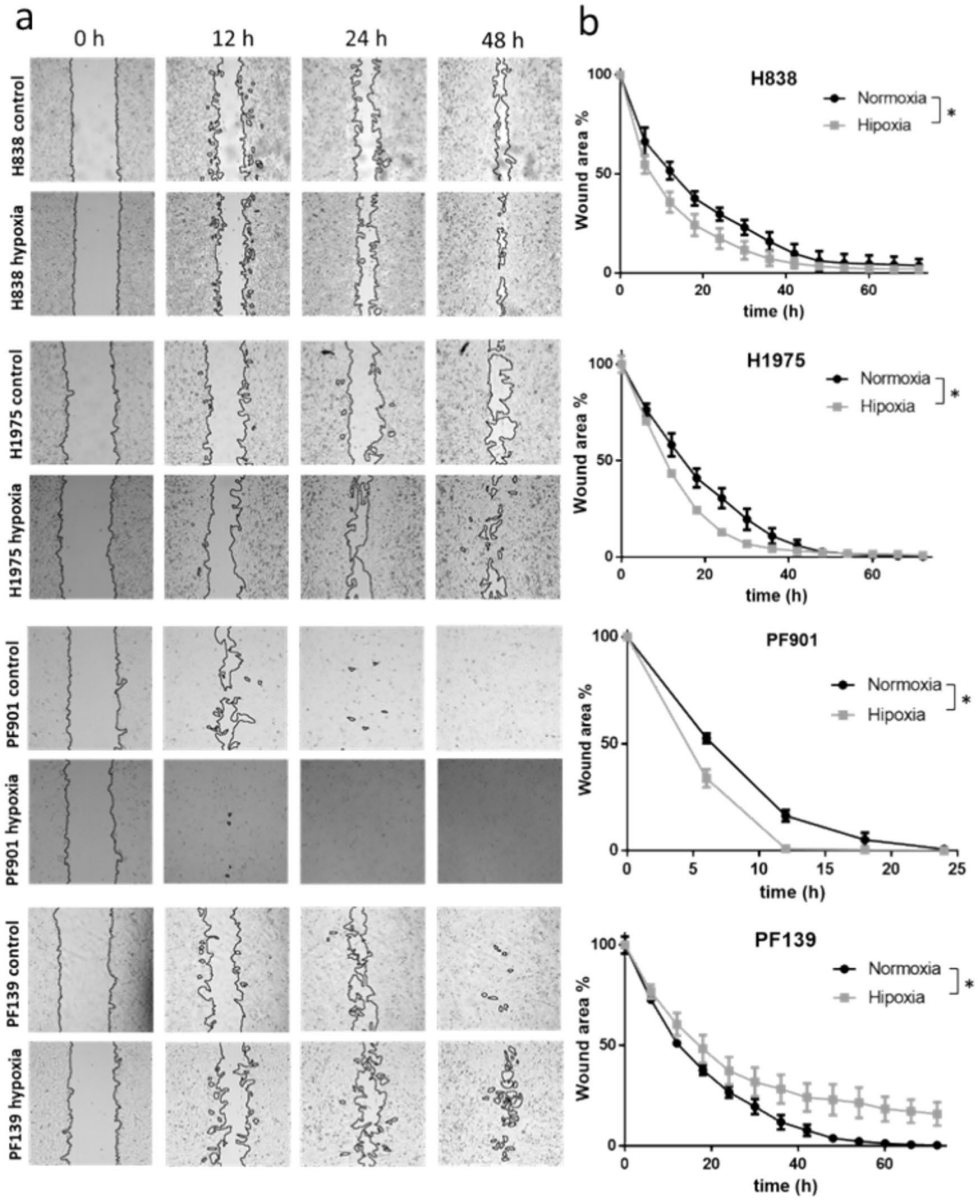
Effects of hypoxia on migratory activity under confluent conditions. Wound closure of the four lung tumor cell lines under normoxic (21% O_2_) and hypoxic (1%O_2_) conditions. a) Representative images of wound closure under normoxic and hypoxic conditions. b) Graphs show normalized data of scratch assay experiments. The images were captured every 6 h on a 72 h time frame. Hypoxia induced faster wound closure in all cell lines except for PF139. Asterisks indicate statistically significant differences at p < 0.05. Data were analyzed using non-linear regression and compared with sum-of-squares F test comparing Hillslope and LogIC50 data. Data are derived from three independent experiments. One independent well was monitored for each experiment. Data plotted as mean ± SEM.

For further analysis of the effect of a hypoxic environment on tumor cells, we characterized migratory activity under confluent conditions using a wound-healing assay under normoxic and hypoxic (1%O_2_) conditions. Notably, hypoxic conditions induced significantly faster wound closure in case of H838, H1975 and PF901 cell lines compared to normoxia. Interestingly, PF139 cell line was not able to completely close the wound under hypoxia, while the scratch was closed under normal oxygen levels within 60 h **(**Fig. 3**).**

### Cell-line dependent changes in the mRNA expression levels and evaluation of EMT markers under hypoxic conditions

To study the molecular response to hypoxia on motility we measured the mRNA levels of main EMT markers, namely vimentin, N-cadherin, and E-cadherin in our panel of lung adenocarcinoma cell lines. Analysis of the basal expression of these genes revealed a slight expression difference between the cell lines for vimentin and E-cadherin. At the same time, both PF139 and PF901 showed significantly higher N-cadherin expression, especially compared to H838 with slow baseline motility **(**Supplementary Fig. 5**)**.

**Fig. 4:**
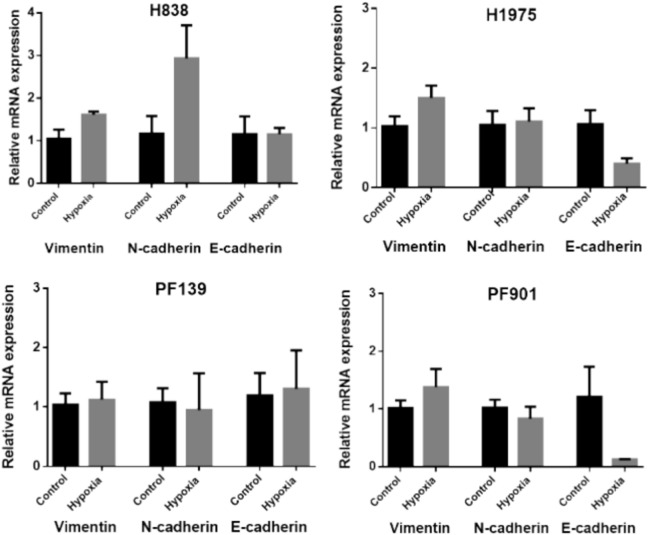
mRNA expression of EMT marker genes under normoxic and hypoxic conditions. Graphs depict the relative mRNA expression values of vimentin, N-cadherin, and E-cadherin obtained by real-time PCR. Cells were exposed for 48 h to normoxic (21% O_2_, control) or hypoxic (1% O_2_) conditions. Hypoxia induced cell line-dependent changes. Data shown was obtained from three independent experiments, plotted as mean ± SEM. RPLP0 was used as the endogenous internal control.

N-cadherin and vimentin levels increased in H838 in both CoCl_2_ treatment and hypoxia, while CoCl_2_ treatment reduced E-cadherin expression (Fig. 4). CoCl_2_ treatment reduced the expression of all genes tested in H1975 **(**Supplementary Fig. 6**)**, while hypoxia slightly increased vimentin expression with a parallel strong reduction of E-cadherin level. In PF139, the levels of vimentin and N-cadherin were slightly increased upon CoCl_2_ treatment **(**Supplementary Fig. 6**)**, but none of the genes were affected by hypoxia. Hypoxia strongly reduced E-cadherin, and slightly increased vimentin level in PF901 cells, while the level of N-cadherin showed no change.

Also, we investigated a number of signaling pathways that was connected with hypoxia and migratory activity previously (Supplementary Fig. 8). Interestingly, phosphorylation of SRC protooncogene, and p38 protein was reduced under hypoxic conditions (Supplementary Fig. 8). Notably, autophosphorylation of focal adhesion kinase (FAK) was only increased in PF139 cell line under hypoxia (Supplementary Fig. 8).

**Fig. 5:**
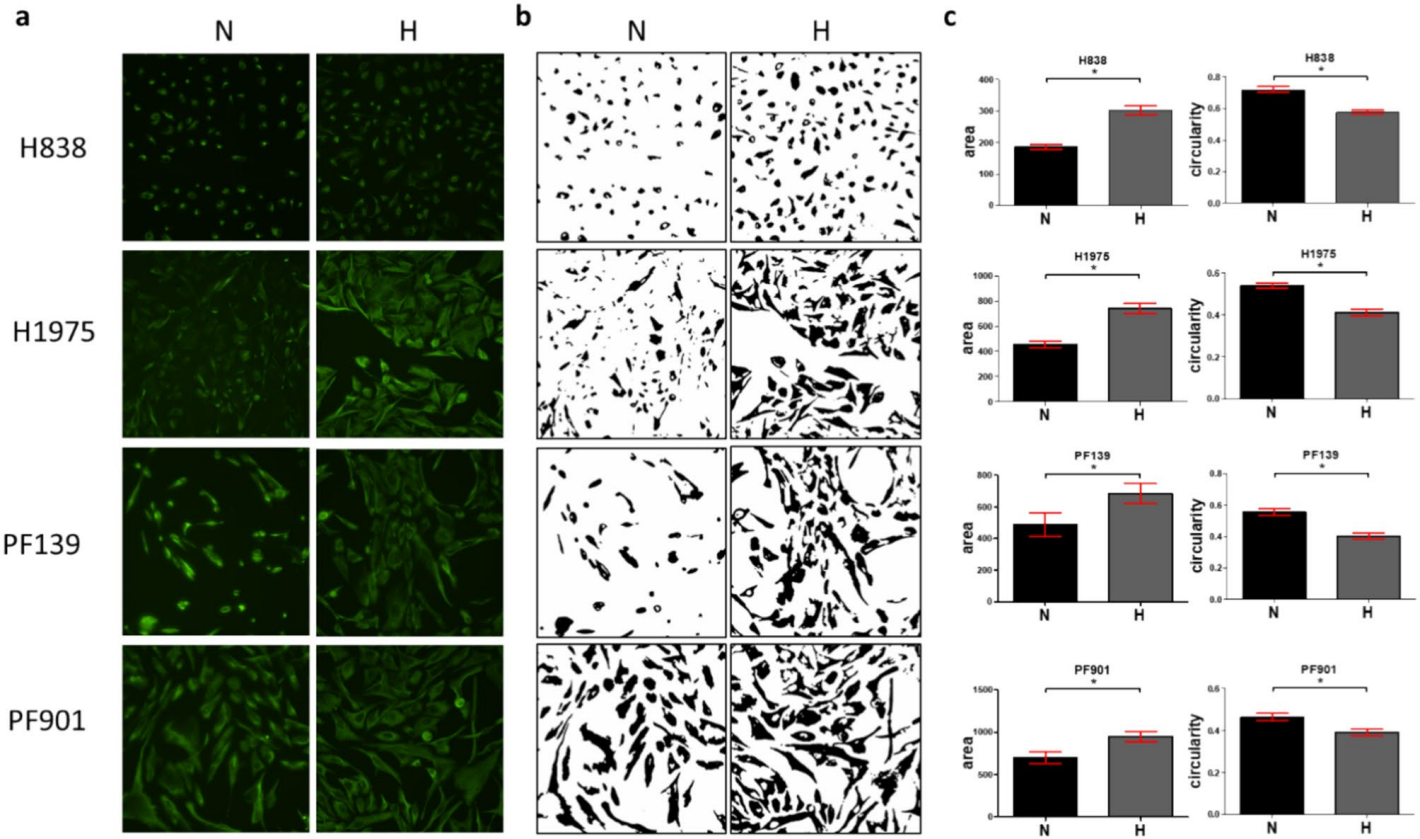
Vimentin expression and morphology under normoxic and hypoxic conditions. (**a**) Pictures show representative images of fluorescently labeled vimentin of lung adenocarcinoma cell lines under normoxic (N) and hypoxic (H) conditions. (**b**) Binary masks created from vimentin expression using ImageJ Fiji software. (**c**) Evaluation of changes in binary masks area and circularity (shape) of the individual cells. Data shown derives from three independent experiments (Mean, + /-SEM). Asterisks marks statistically significant differences. Significant differences were established using Mann-Whitney U test at p < 0.05.

Next, we asked whether the intracellular distribution of vimentin, a key intermedier filament protein connected to mesenchymal phenotype, changes upon hypoxia. To address this, we performed immunofluorescence analysis, where we observed that vimentin showed uneven distribution, often localized around the nucleus under normoxic conditions. However, vimentin filaments drastically changed morphology with even distribution fitting the shape of the cells after incubation with low oxygen concentrations. (Fig. 5a) Notably, area of vimentin expression in the individual cells were significantly larger under hypoxic conditions. Also, shape of the binary masks of vimentin signal showed lower circularity under hypoxic conditions, indicating a shift towards a more mesenchymal phenotype (Fig. 5b,c). Notably, these findings are in accordance with previous reports describing a EMT-related redistribution of vimentin in various cell types^21,22^.

## Discussion

Lung cancer is the most deadly form of malignant diseases globally. This malignancy is associated with a poor prognosis due to late diagnosis of an already advanced stage of metastatic disease^23,24^. Metastasis is a process whereby malignant cells leave the primary tumor, infiltrate neighboring tissues, enter the circulatory system, and establish secondary tumors in distant organs. Metastasis is the leading cause of mortality in lung cancer patients^4^, making it crucial to understand the underlying processes. Hypoxia, an environmental condition that is commonly found in tumors due to the unmatured vascularization, characterized by low levels of oxygen, has been associated with the progression of tumor and the development of metastasis^5^. Investigating the effects of tumor hypoxia in clinical setting is a challenging process complexified by tumor heterogeneity. Accordingly, we addressed tumor heterogeneity by investigating cells harboring major mutations that are commonly found in lung adenocarcinoma, utilizing H1975 (EGFR L858R and T790M), PF901 (BRAF V600E), PF139 (KRAS G12C), and H838 (triple wild type with no known driver mutation) cell lines.

Detailed scientific investigation of hypoxia has revealed that hypoxia inducible factors (HIFs) are the major regulatory proteins mediating cellular response to low oxygen conditions. Notably, previous research has indicated that elevated levels of HIF-1α and HIF-2α in non-small cell lung cancer (NSCLC) are correlated with an unfavorable prognosis for patients^25,26^. However, in certain malignancies, elevated HIF-1α levels are associated with lower cancer stage or decreased patient mortality, highlighting context-dependent functions for HIF-1α^27^. Stabilization of HIF occurs in most cases as a response to hypoxic conditions, therefore the examination of this protein plays a central role in hypoxia research^28^. Indeed, we observed dramatically high expression of HIF-1α protein after 48 h exposure to hypoxic conditions. Although HIF-1 is crucial in hypoxic response, many studies only focus on the stabilization of HIF-1α by chemical agents rather than achieving real hypoxic conditions. An example of hypoxia mimicking agents is CoCl_2_, one of the most commonly used HIF-1α stabilizer, which, in our case, induced higher HIF-1α protein level in all cell lines compared to hypoxic conditions. Nevertheless, no equation should be made between hypoxia and HIF stabilization. Despite this, a wide range of diverse and sometimes contradictory effects of “hypoxia” have been described in in vitro studies based on hypoxia mimicking agents. Thus, strictly validated studies investigating effects of hypoxia on tumor progression is urgently needed.

In order to obtain a general overview of how hypoxia affects cells, we first examined changes in the protein levels of the proliferation marker p-Histone H3 and the level of cleaved PARP, indicating apoptosis induction. Interestingly, we noted reduction of p-Histone H3 levels in most of the cell lines which suggests hypoxia to be a cellular stressor in this tested time exposure. In the meantime, no change could be observed in the proliferation of tumor cells under hypoxic conditions using a direct viability test. Our findings are in accordance with prior studies suggesting that reduced oxygen concentrations may either decrease^29^ or exhibit no impact on the proliferation of various tumor cells^30^. Notably, we found no changes in the level of cleaved PARP compared to normoxic condition, indicating that hypoxia does not induce apoptosis in this panel of lung adenocarcinoma cell lines.

However, the most significant effects of tumor hypoxia are thought to be related to tumor cell migration and metastasis and most of the studies focus on the hypoxia-induced pro-motility effects^31–33^, accordingly, we also investigated cellular motility via time-lapse videomicroscopy. In a previous study, we demonstrated that the HT168-M1 melanoma cell line exhibited increased migration and metastasis formation under hypoxia, indicating heightened aggressiveness^20^. Lehmann and colleagues found that HIF stabilization led to an increased number of metastatic events in an in vivo lung colonization model with murine breast cancer^34^. To our surprise, we observed that single-cell haptotactic motility was inhibited in 3 out of 4 cell lines upon 48 h of exposure to hypoxic conditions. To present, there is limited research available on the effects of in vitro hypoxia on the migratory activity of lung adenocarcinoma cell lines. However, hypoxia or hypoxia mimicking agents generally enhances the migration of lung tumor cell lines, although none of them were investigated the single-cell motility utilizing time-lapse video microscopy^33,35–37^. Additionally, most of these studies are focusing on the motility of A549 cell line. However, a recent study revealed heightened cellular motility in the H838 cell line following a 24 h exposure to hypoxia^37^. In accordance with this result, we also found increased migratory activity in the initial 24 h time interval for H838 cells.

As tumor cells may use numerous strategies for local invasion utilizing distinct types of migration, we also investigated invasion of tumor cells by scratch assay. Notably, most of the studies investigating hypoxia effects utilize this motility assay for evaluation of tumor migration. Surprisingly, we found that hypoxia significantly enhanced wound closure in three out of four cell lines including H838 and PF901 cell lines, that exhibited reduced single cell motility in hypoxic conditions. Accordingly, we sought to examine the underlying molecular mechanisms, investigating changes in the EMT markers that may contribute to the observed effects.

Notably, we observed significant differences in the basal expression of N-cadherin in our cell panel, that showed significantly higher expression in two cell lines. Of note, literature suggests that N-cadherin may promote motility and invasion of tumor cells and its overexpression is associated with shorter overall survival in non-small cell lung cancer patients^38^.

Interestingly, we observed changes in the expression of EMT markers in our cell panel that were consistent with the metastasis-promoting effect of hypoxia. These included decreased E-cadherin expression under hypoxic conditions in PF901 and H1975 cell lines and increased N-cadherin expression in H838. No changes were observed in EMT markers in PF139, the only cell line that exhibited slower migratory activity in scratch assay upon hypoxic conditions. Furthermore, this was the only cell line that showed increased FAK autophosphorylation in hypoxic conditions (Supplementary Fig. 7). This finding may indicate interference with the dynamic assembly-disassembly of focal adhesions as FAK activation is indispensable for both event, therefore it is crucial for proper migratory activity^39^. Interestingly, there is growing evidence for a tumor suppressor role of HIF-1alpha in certain cases^40,41^. Furthermore, a recent study has shown that ablation of HIF-1α in KRAS G12D mice resulted in increased invasion, metastasis formation and decreased survival^42^. The fact that PF139 also harbors KRAS mutation may also raise interesting questions about the association of oncogenic KRAS and HIF1α in tumor progression.

Last, we also investigated vimentin expression using immunofluorescent staining. In line with RNA data described above, we found that hypoxia exerted pronounced effects on vimentin morphology. In normoxia, vimentin showed uneven, varying distribution within cell boundaries, many times localized near to the nucleus. However, under hypoxic conditions, vimentin exhibited more homogenic, filamentous distribution in all cell lines that showed more mesenchymal morphology. Notably, similar changes were described previously on various cell types by EMT induction using TGF-β^22^.

Overall, we demonstrated cell-line and time-dependent effects of hypoxia in cell migration. Notably, hypoxia either not changed or slightly reduced cellular proliferation, based on viability tests and investigation of proliferation markers. Notably, we found that hypoxia mimicking CoCl_2_ failed to phenocopy effect of hypoxic conditions on single-cell motility. Importantly, we found that invasion under confluent conditions using scratch assay was enhanced in hypoxia in most of the cell lines, even in those that exhibited lower migratory activity in single-cell tracking in hypoxic conditions. In addition, we found that changes in EMT markers were in line with enhanced migration observed in scratch assay. Although the exact mechanism underlying different effect of hypoxia on single cell motility and wound closure is yet to be fully elucidated, distinct pattern of cell–cell and cell-ECM contacts is probably a major influencing factor, probably through modulation of transition from epithelial to mesenchymal phenotype.

Our study gives important insight into hypoxia- and HIF-related response of a wide variety of common lung adenocarcinoma models in terms of motility and proliferation, as well as apoptotic activity. As the high variance of cellular response might be the major underlying treatment obstacle, leading to failure of clinical use of HIF-inhibitors, further studies are warranted for better understanding of impact of hypoxia on cancer progression^23^.

### Limitations

As most of the scientific works, this study also has some limitations. 3D migrational methods and in vivo metastatic studies are of higher relevance and more closely predict distinct steps of the metastatic cascade. However, this study only utilized 2D in vitro experiments, single cell motility and scratch assay, for migrational studies. Interestingly, these methodologies revealed differences between single-cell migration and invasion under confluent conditions, however, the mechanistic results obtained in our experiments do not allow us to distinguish between single and confluent cells. This limitation makes it challenging to precisely attribute the observed phenomena to specific cellular EMT gene and protein expression.

## Methods

### Cell culture

Human lung adenocarcinoma cell lines PF901, PF139, H838 and H1975 were cultured in DMEM (Dulbecco’s Modified Eagle Medium; Lonza, Basel, Switzerland); containing 4500 mg/dm^3^ glucose, pyruvate and L-glutamine) supplemented with 10% FBS (Fetal Bovine Serum; EuroClone, Pero, MI, Italy), 1% antibiotic–antimycotic solution (Lonza) and incubated at 37 °C in a humidified 5% CO_2_ atmosphere. For hypoxia measurements, cells were cultured in 1% O_2_, 5% CO_2_ and 94% N_2_ to stimulate the hypoxic environment. H838 cells were purchased from Horizon Discovery Ltd, while H1975 cells derived from ATCC. PF139 and PF901 cells were established from malignant pleural effusion samples in cooperation with the West German Biobank Essen as described earlier^43,44^. The patients provided written informed consent and the experiments were approved by the Ethics Committee of the University Hospital Essen (#18-8208-BO). Otherwise, this study does not include experimental animals or human participants. All experiments were performed in accordance with relevant guidelines and regulations.

### Videomicroscopy

Videomicroscopy was performed with 4000 cells/well seeding concentration in 24-well cell culture plates (Greiner AG, Kremsmünster, Austria). The day after the cells were plated, medium was replaced with fresh medium with or without 200 µM CoCl_2_ (Sigma-Aldrich, St. Louis, Missouri, USA). Preliminary experiments had shown that no cytotoxicity occurred at this concentration. Three independent wells were used for each treatment group (normoxia; normoxia + CoCl_2_; hypoxia). One field of view per well was imaged every 10 min during the 48 h treatment period using a zenCellowl incubator microscope (innoME, Espelkamp, Germany).

For cell migration, videos were analyzed using CellTracker_v1_1_1 Standalone Version free software available at http://celltracker.website/index.html. Migratory activity of cells was measured using the semi-automatic tracking function of the software (Vignetting correction settings: Bicubic interpolation; Block size = 100; Maximum allowed displacement = 20; Semi-automatic tracking settings: Template matching; Maximum allowed displacement = 20; Cell diameter = 50). The resulting trajectories were checked and corrected if necessary using the manual function of the software. Following this step, data generated by the software were exported to an excel spreadsheet for further analysis. Results from three independent experiments were used for the calculations. Data were divided into 12 h intervals (1–72, 73–144, 145–216, 217–288 frames) and were further analyzed using the free software Chemotaxis and Migration Tool (Ibidi, Gräfelfing, Germany). Briefly, distance from origin and total length data were obtained from trajectories of the cells that remained in the field of view through the given time interval. The results were plotted and statistically evaluated using GraphPad Prism 5 software (La Jolla, San Diego, CA, USA). Representative videos can be found at the supplementary videos.

### Cytotoxic sulforhodamine-B (SRB) assay

In brief, the cells were seeded in a 96-well plate at a concentration of 1 × 10^2^ or 1 × 20^3^ cells/well in 200 µL fresh DMEM (Lonza) supplemented with 10% FBS (EuroClone), 1% antibiotic–antimycotic solution (Lonza) and left to attach to the plates for 24 h. Then, cells were incubated at 37 °C in a humidified 5% CO_2_ atmosphere or for hypoxic conditions, cells were incubated in 1% O_2_, 5% CO_2_ and 94% N_2_ for 48 h. After that, the cells were washed and fixed with 100 µL cold 10% Trichloroacetic acid (TCA) for 1 h at 4 ℃. The wells were then washed 2 times with distilled water and stained for 15 min at room temperature with 70 µL 0.4% SRB dissolved in 1% acetic acid. Then, cells were washed with 1% acetic acid 3 times. The plates were air-dried and the dye was solubilized with 200 µl/well of 10 mM tris base (pH 10.5) for 10 min. The optical density (O.D.) of each well was measured spectrophotometrically at 570 nm with BioTek 800 microplate reader (Agilent, Santa Clara, CA) with automatic shaking for 30 s before reading. The mean background absorbance was subtracted automatically and mean values for each conditions were calculated.

### Scratch assay

Scratch assays were prepared using the MuviCyte™ live-cell Imaging System (PerkinElmer, Waltham, MA, USA) supplied with a MuviCyte™ scratcher (PerkinElmer) to perform standardized scratches on 96 well plates following the manufacturer’s instructions. Briefly, cells were seeded at 40.000 cells/well density and left for overnight to attach. The next day, scratches were made using the MuviCyte™ scratcher device and wells were washed with DPBS to remove debris. DPBS was replaced with fresh medium. Wound closure was monitored in four parallel wells for each cell line for 72 h period. Pictures were taken from every well in every 6 h. Analysis of wound closure were performed using a modified script from Suarez-Arnedo, A. and colleges^45^ for ImageJ Fiji software. The script automatically measures the area of the scratch for different time points. Data were normalized and expressed as percentage of the starting scratch area. For each experiment, data from the four independent wells for each cell line were averaged and used as a single replicate, graphed in GraphPad Prism 5 software. Three independent biological replicates were performed. T_1/2_ wound closure data was calculated with GraphPad Prism 5 software using nonlinear regression (log [inhibitor] vs normalized response, variable slope). Representative videos can be found at the supplementary materials.

### Western blot

The effects of CoCl_2_ treatment or hypoxia on protein expression and activation were investigated by immunoblot analysis. Cells were plated on 6-well plates (Greiner AG) at 200.000 cell/well density and were exposed to normoxia, normoxia + 200 µM CoCl_2_ treatment or hypoxia for 48 h. At the end of the treatment period, cells were washed with DPBS and were fixed with 6% trichloroacetic acid for 1 h at 4 °C. Cells were then mechanically scraped and centrifuged at 6000 RCF for 15 min. The precipitated protein was dissolved in a modified Läemmli-type buffer containing 0.02% bromophenol blue, 10% glycerol, 2% SDS, 100 mM dithiothreitol (DTT), 5 mM EDTA, 125 mg/ml urea, 90 mM Tris–HCl, pH 7.9. A Qubit fluorometer (Invitrogen™ Waltham, Massachusetts, USA) was used to determine protein concentration. Following this step, equal amounts of protein (20 µg/lane) from each sample were loaded to a 10% polyacrylamide gel and blotted onto PVDF membranes after electrophoretic separation using 10% precast polyacrilamide gels and Turbo-Blot system (BIO-RAD). Hypoxic response was evaluated by assessing changes in the protein level of HIF-1α (D5F3M, Cell Signaling Technology Danvers, MA, US), PARP (9542, Cell Signaling Technology),p-Histone H3 (9701S, Cell Signaling Technology), p-FAK (3283 Cell Signaling Technology), FAK (3285 T, Cell Signaling Technology), p38 (8690S, Cell Signaling Technology), p-p38 (4511, Cell Signaling Technology) (primary antibodies were used to detect changes in apoptosis and cell proliferation, respectively. β-tubulin (2128 T, Cell Signaling Technology) primary antibody was used as the internal control. All antibodies were dissolved in 5% BSA (bovine serum albumin) or nonfat dry milk in 1 × TTBS (Tris-buffered saline supplemented with 1% Tween80) buffer according to the manufacturer’s instructions. Membranes were blocked at room temperature (RT) for one hour in 5% milk dissolved in 1 × TTBS and incubated overnight at 4 °C with primary antibodies. HRP-conjugated rabbit secondary antibody (1:10000, 1 h, RT) and Pierce ECL Western Blotting Substrate (Thermo Fisher Scientific, Waltham, MA, USA) were used for signal development. Ponceau total protein staining was used for normalization. Quantitation was performed using ImageLab (Bio-Rad Hercules, CA, USA) software. Densitometric evaluations were always performed on the original blots. For the figures in the article, brightness and contrast adjustments were made for better visualization. All four cell lines were analyzed in 3 biological replicates. Uncropped original blots can be found at supplementary info file.

### Real-time reverse transcription PCR

Samples were isolated in TRIzol reagent (Ambion, Life Technologies, Carlsbad, CA, USA) and the Direct-zol™ RNA MiniPrep (Zymo Research) kit was used to isolate RNA according to the manufacturer’s protocol. The concentration and purity of the RNA were determined using spectrophotometry at 260/280 and 260/230 nm (NanoDrop One, ThermoFisher Scientific, Madison, WI, USA). The RNA was then reverse transcribed using a Reverse Transcription System (Promega Corporation, Madison, WI, USA) according to the manufacturer's instructions. The resulting cDNA samples were stored at −80 °C until further use. Quantitative real-time PCR reactions were performed using SsoAdvanced Universal SYBR Green Supermix (Bio-Rad, Hercules, CA, USA) in a CFX96™ Real-Time PCR System according to the manufacturer’s instructions. The following target genes were studied: vimentin (F: 5′–AGTCCACTGAGTACCGGAGAC and R: 5′-CATTTCACGCATCTGGCGTTC), E-Cadherin (F: 5′–ATTTTTCCCTCGACACCCGAT and R: 5′-TCCCAGGCGTAGACCAAGA), N-Cadherin (F: 5′–AGCCAACCTTAACTGAGGAGT and R: 5′-GGCAAGTTGATTGGAGGGATG). RPLPO (F: 5′–AGCCCAGAACACTGGTCTC and R: 5′-ACTCAGGATTTCAATGGTG) was used as the internal control. The mRNA expression was analyzed by relative quantification, threshold cycle (C_t_) values and 2^−ΔΔCt^ method.

### Immunofluorescent analysis

For immunofluorescent analysis, cells were seeded on glass immunohistological slides in 10% FBS + DMEM droplets. Next day, scratches were made in all colonies and then cells were cultured for 48 h in normoxic or hypoxic conditions, then were fixed for 20 min in 4% formaldehyde. Cells were then repeatedly washed with PBS, then permeabilized with 0.01% TritonX100 for 5 min. Following additional washing steps after permeabilization, cells were incubated with vimentin primary antibody Clone V9 (Agilent, USA) for an hour at RT according to the manufacturer’s instructions, then were washed in PBS and probed for half an our with anti-Mouse IgG (H + L) Secondary Antibody, Alexa Fluor™ 488 (A-11029, Thermo Fisher Scientific, USA). Nuclei was stained with DAPI, and slides was covered with ProLong Gold antifade reagent (P36930, Thermo Fisher Scientific), then were visualized using an Eclipse E600 (Nikon Optoteam, Vienna, Austria) fluorescent microscope with a CCD camera.

Pictures of cells were analyzed in ImageJ Fiji open-source software. Briefly, a top-hat filter were applied (50 px radius), then resulting pictures were convert to binary mask. “Median” command of binary transformations were applied (3 px radius), then particles (binary representatives of vimentin expression of each cells) were analyzed (Particle size: 100–4000 pxs). Results were manually revised and segmented if necessary, based on the original fluorescent images. Area and circularity data were collected from three independent experiments and were graphed with GraphPad Prism 5 software.

### Statistical analysis

Statistical analysis were performed by Graphpad Prism 5 software. Briefly, single-cell motility data were analyzed for normal distribution using Shapiro-Wilk test. As number of data points were too small for Shapiro-Wilk test in case of proliferation data, we employed the Kolmogorov-Smirnov test to examine the normal distribution for this dataset. Datasets with normal distribution were analyzed by ANOVA followed by Tukey’s Multiple Comparison test, otherwise Kruskal-Wallis test were applied followed by Dunn’s Multiple Comparison test. Data from scratch assays were analyzed using non-linear regression and compared with sum-of-squares F test comparing Hillslope and logIC_50_ data. RNA and protein expression data was analyzed using unpaired t-test with Welch correction.

## Supplementary Information

Supplementary Figures.

Supplementary Information.

Supplementary Video 1.

## Data Availability

All data acquired in experiments are either presented in this study or are available at the corresponding author upon reasonable request.
