# Supplementary material for: Differential effects of hypoxia on motility using various in vitro models of lung adenocarcinoma
Source: Sci Rep. 2024 Sep 3;14:20482. doi: 10.1038/s41598-024-70769-w (PMC11372077; doi:10.1038/s41598-024-70769-w)
Supplement: Supplementary file 1 — Supplementary Figures. [file 41598_2024_70769_MOESM1_ESM.pdf]

Title: Differential effects of hypoxia on motility using various in vitro models of lung adenocarcinoma

Author list: Sára Eszter Surguta, Marcell Baranyi, Laura Svajda, Mihály Cserepes, Ivan Ranđelović, Enikő Tátrai, Balázs Hegedűs, József Tóvári

Supplementary Figures

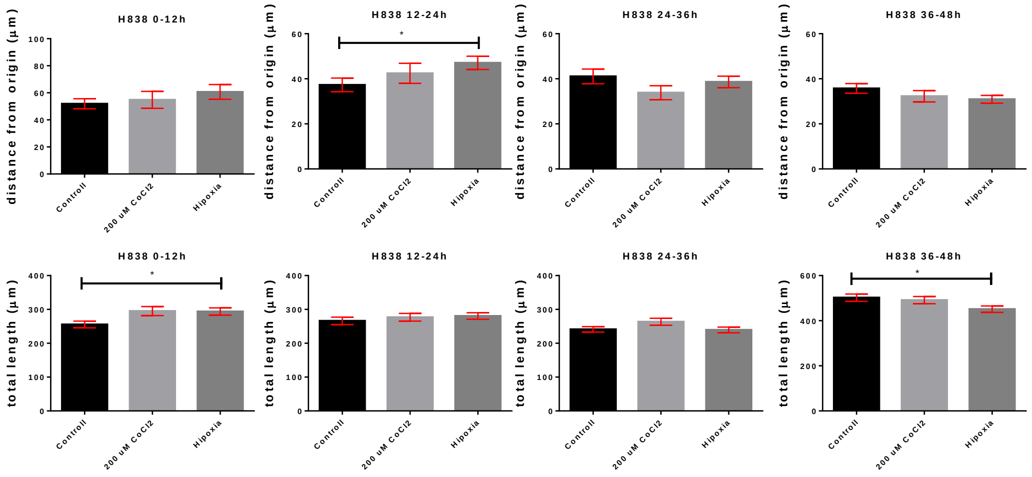

Supplementary figure 1. Motility of individual cells in 12-hour intervals of the 48-hour-long exposure to normoxia (21% O_2), 200 µM $CoCl_2$ treatment or hypoxia (1% O_2). Only cells that were presented on the video for the whole time period were included in the analyses. The distance from the origin and the total length are plotted. Asterisks marks statistically significant differences. Data is derived from three independent experiments. Two independent wells were monitored for each experiment for normoxia and 200 µM $CoCl_2$ treatment, while four independent wells were monitored for hypoxia. Normality was tested with the Shapiro-Wilk test. If the normal distribution was confirmed, one-way ANOVA followed by Tukey’s Multiple Comparison test, otherwise Kruskal-Wallis test was used followed by Dunn’s multiple comparison post-test. Data plotted as mean +/- SEM.

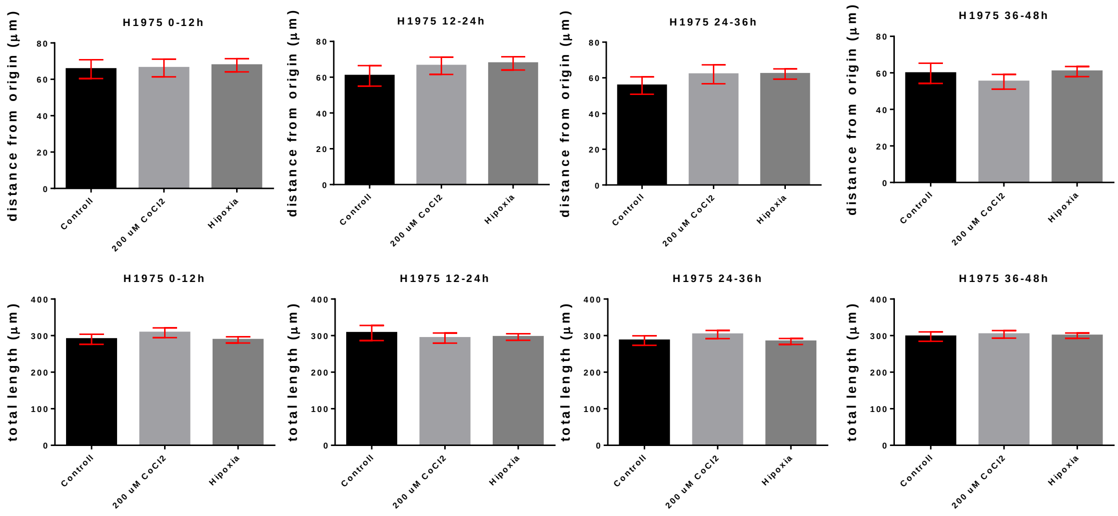

Supplementary figure 2. Motility of individual cells in 12-hour intervals of the 48-hour-long exposure to normoxia (21% O_2), 200 µM $CoCl_2$ treatment or hypoxia (1% O_2). Only cells that were presented on the video for the whole time period were included in the analyses. The distance from the origin and the total length are plotted. Asterisks marks statistically significant differences. Data is derived from three independent experiments. Two independent wells were monitored for each experiment for normoxia and 200 µM $CoCl_2$ treatment, while four independent wells were monitored for hypoxia. Normality was tested with the Shapiro-Wilk test. If the normal distribution was confirmed, one-way ANOVA followed by Tukey’s Multiple Comparison test was applied, otherwise Kruskal-Wallis test was used followed by

Dunn's multiple comparison post-test. Data plotted as mean +/- SEM.

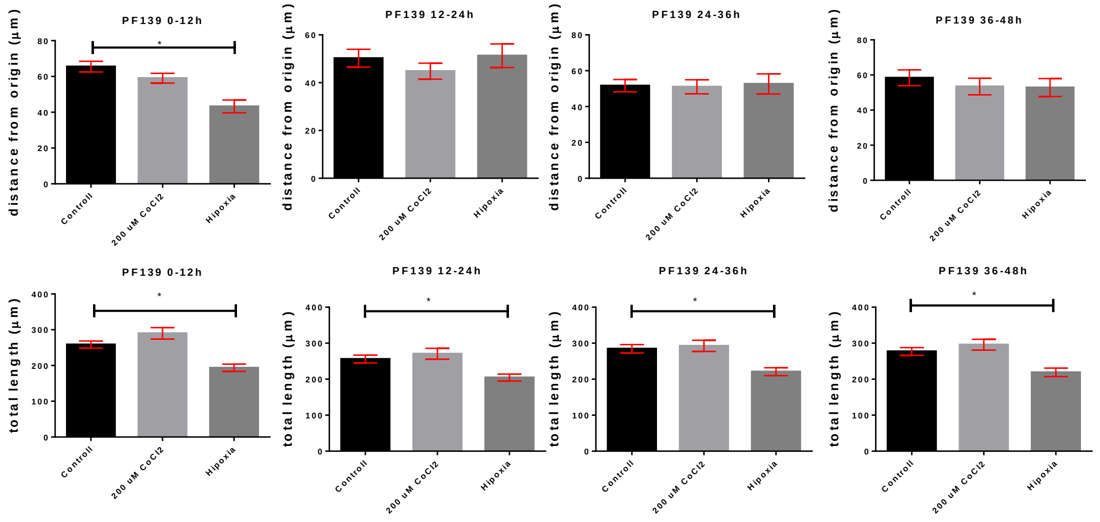

Supplementary figure 3. Motility of individual cells in 12-hour intervals of the 48-hour-long exposure to normoxia (21% O_2), 200 µM $CoCl_2$ treatment or hypoxia (1% O_2). Only cells that were presented on the video for the whole time period were included in the analyses. The distance from the origin and the total length are plotted. Asterisks marks statistically significant differences. Data is derived from three independent experiments. Two independent wells were monitored for each experiment for normoxia and 200 µM $CoCl_2$ treatment, while four independent wells were monitored for hypoxia. Normality was tested with the Shapiro-Wilk test. If the normal distribution was confirmed, one-way ANOVA followed by Tukey's Multiple Comparison test was applied, otherwise Kruskal-Wallis test was used followed by Dunn's multiple comparison post-test. Data plotted as mean +/- SEM.

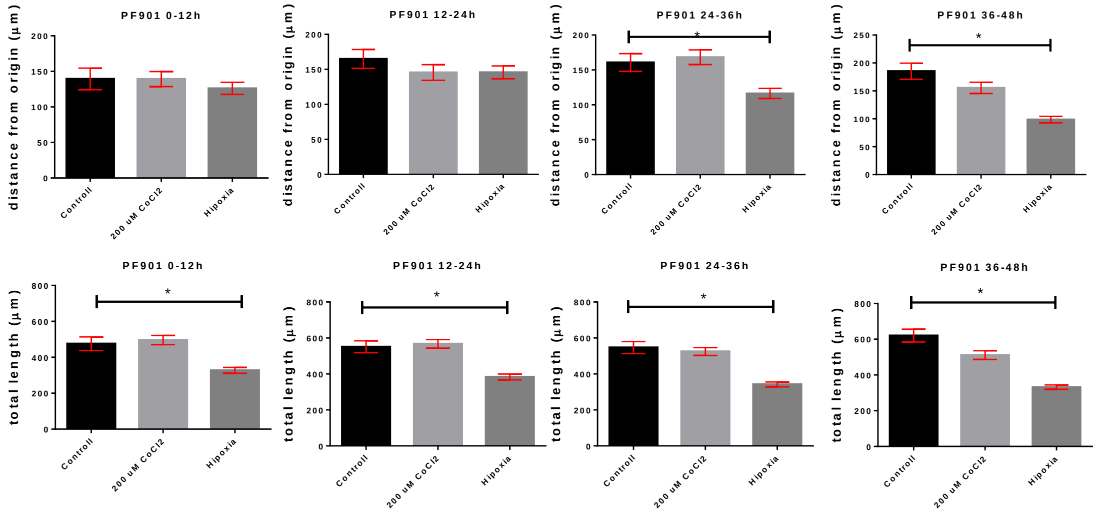

Supplementary figure 4. Motility of individual cells in 12-hour intervals of the 48-hour-long exposure to normoxia (21% O_2), 200 µM $CoCl_2$ treatment or hypoxia (1% O_2). Only cells that were presented on the video for the whole time period were included in the analyses. The distance from the origin and the total length are plotted. Asterisks marks statistically significant differences. Data is derived from three independent experiments. Two independent wells were monitored for each experiment for normoxia and 200 µM $CoCl_2$ treatment, while four independent wells were monitored for hypoxia. Normality was tested with the Shapiro-Wilk test. If the normal distribution was confirmed, one-way ANOVA followed by Tukey's Multiple Comparison test was applied, otherwise Kruskal-Wallis test was used followed by Dunn's multiple comparison post-test. Data plotted as mean +/- SEM.

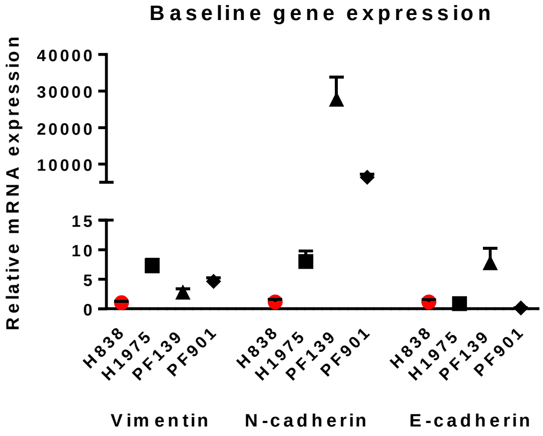

Supplementary figure 5. Baseline expression level of genes involved in epithelial-mesenchymal transition. Relative mRNA expression values of baseline expression under normoxic conditions normalized to the basal expression of H838 measured by qPCR show differences in gene expression levels between the four lung adenocarcinoma cell lines. Data plotted as mean ± SEM.

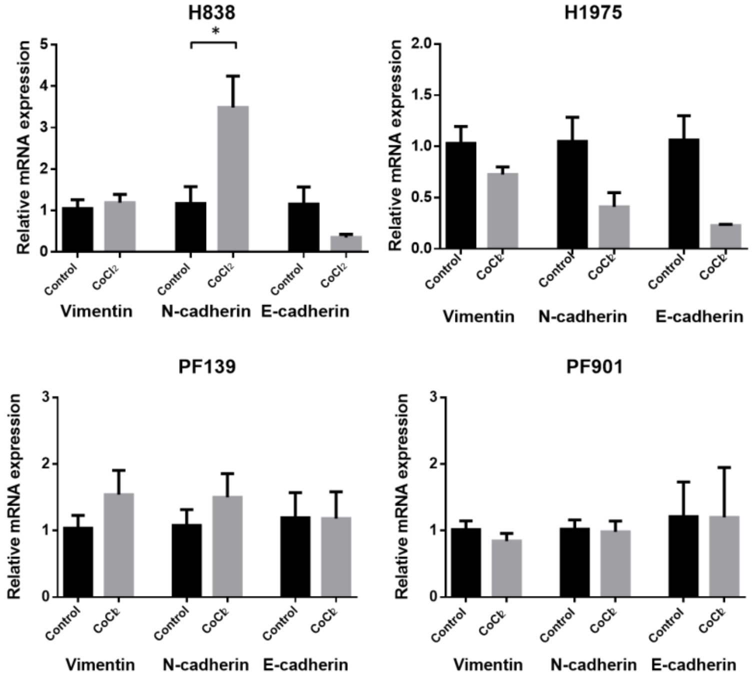

Supplementary figure 6. mRNA expression of EMT marker genes under normoxic and $CoCl_2$ treated conditions. Graphs depict the relative mRNA expression values of Vimentin, N-cadherin, and E-cadherin obtained by real-time PCR (data are mean ± SD, n=3). Cells were exposed for 48 hours to normoxic (21% O_2, control) or 200 µM $CoCl_2$ treatment (21% O_2). $CoCl_2$ induced cell line-dependent changes. Data shown was obtained from three independent experiments, plotted as mean +/- SEM. Asterisks show statistically significant differences. Statistical significance was established using unpaired t-test with Welch correction at $p<0.05$. RPLP0 was used as the internal control.

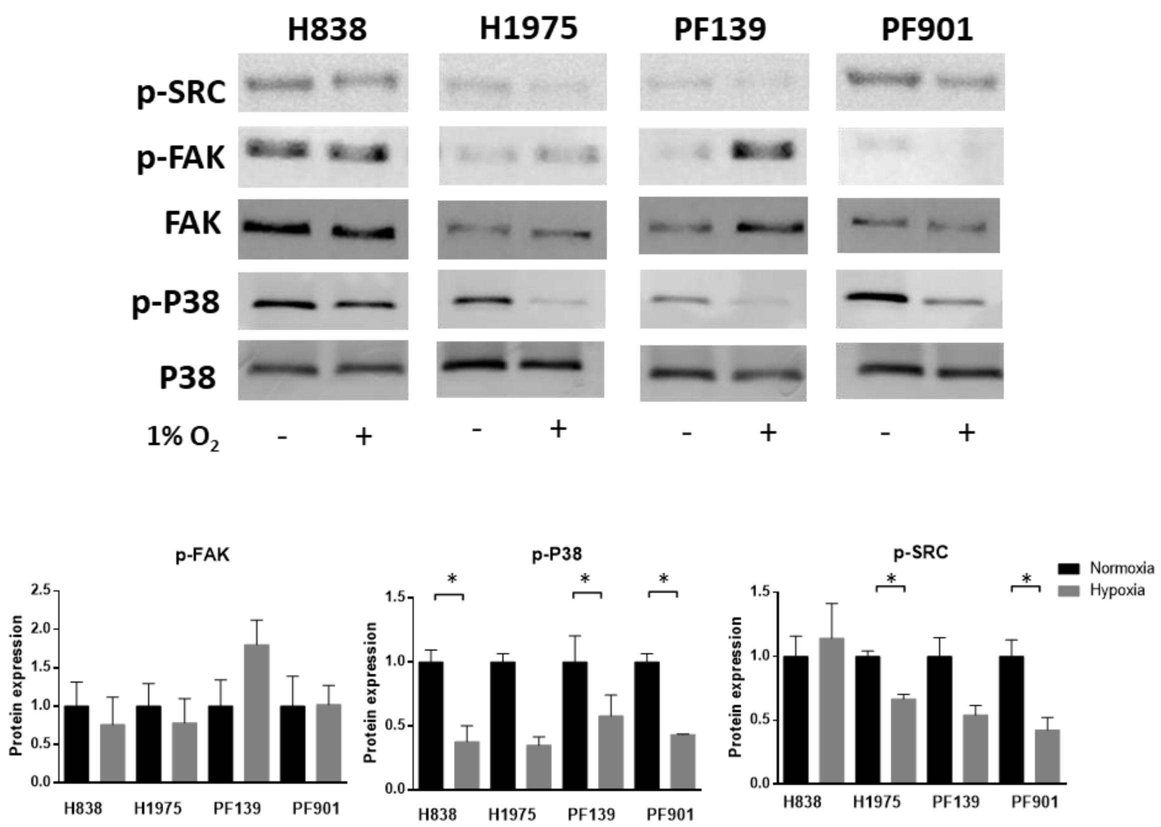

Supplementary figure 7. Effect of hypoxia on distinct related signaling pathways. Cells were exposed for 48 hours to normoxic (21% O_2, control) or hypoxic (1 % O_2) conditions. Representative Western blot images showing protein level changes upon hypoxic conditions. Asterisks show statistically significant differences. Statistical significance was established using unpaired t-test with Welch correction at $p<0.05$.
