# Supplementary material for: Differential effects of hypoxia on motility using various in vitro models of lung adenocarcinoma
Source: Sci Rep. 2024 Sep 3;14:20482. doi: 10.1038/s41598-024-70769-w (PMC11372077; doi:10.1038/s41598-024-70769-w)
Supplement: Supplementary file 2 — Supplementary Information. [file 41598_2024_70769_MOESM2_ESM.pdf]

Differential effects of hypoxia on motility using various in vitro models of lung adenocarcinoma

Author list: Sára Eszter Surguta, Marcell Baranyi, Laura Svajda, Mihály Cserepes, Ivan Ranđelović, Enikő Tátrai, Balázs Hegedűs, József Tóvári

In the Western Blot experiment, following blotting, the membranes were stained with Ponceau to visualize total protein levels for normalization. Subsequently, post-stain removal, the membrane was precisely sectioned according to molecular marker positions and the expected protein sizes. Thus, development of chemiluminestent signal on films show the segmented membrane pieces with the corresponding protein levels.

Total protein with Protein MW marker (5-245 kDa) (Prestained ProteinSHARP*MASS*™ VI, EuroClone S.p.A., 6 Pero, Italy)

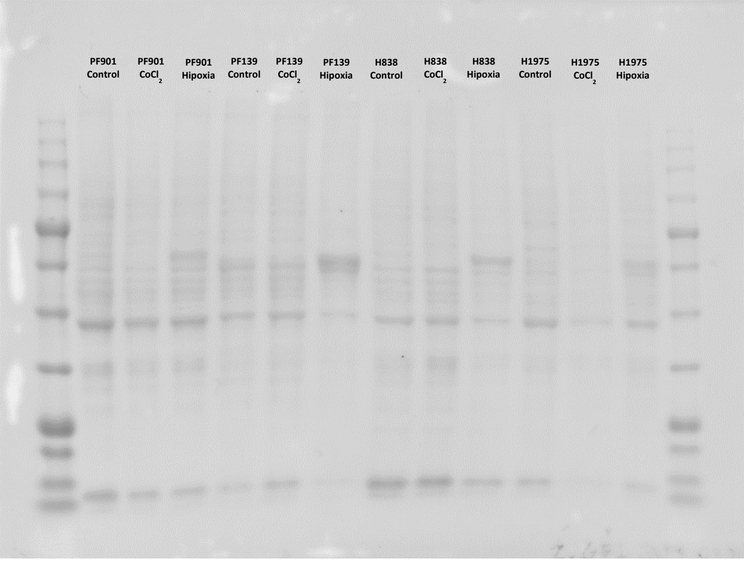

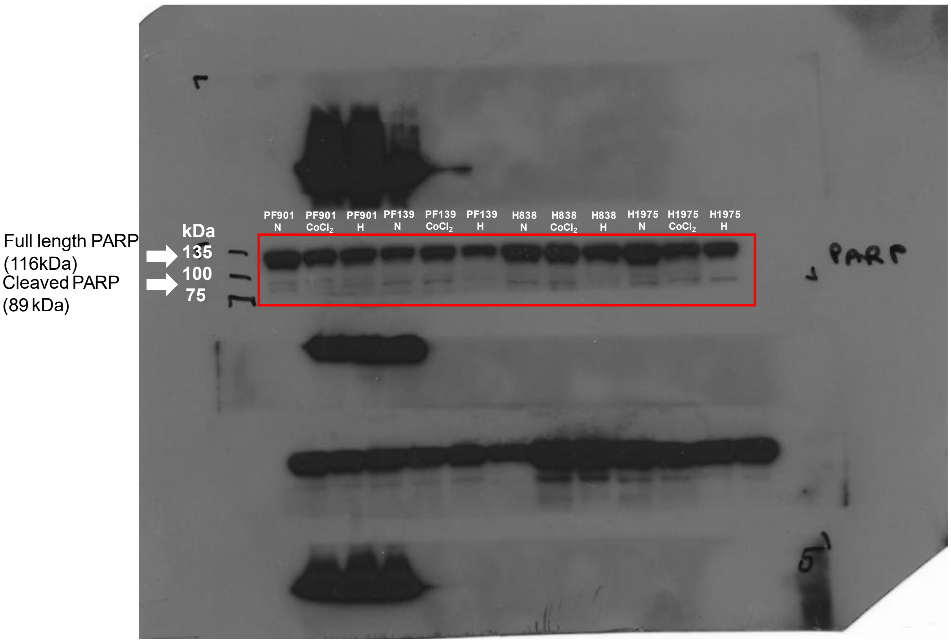

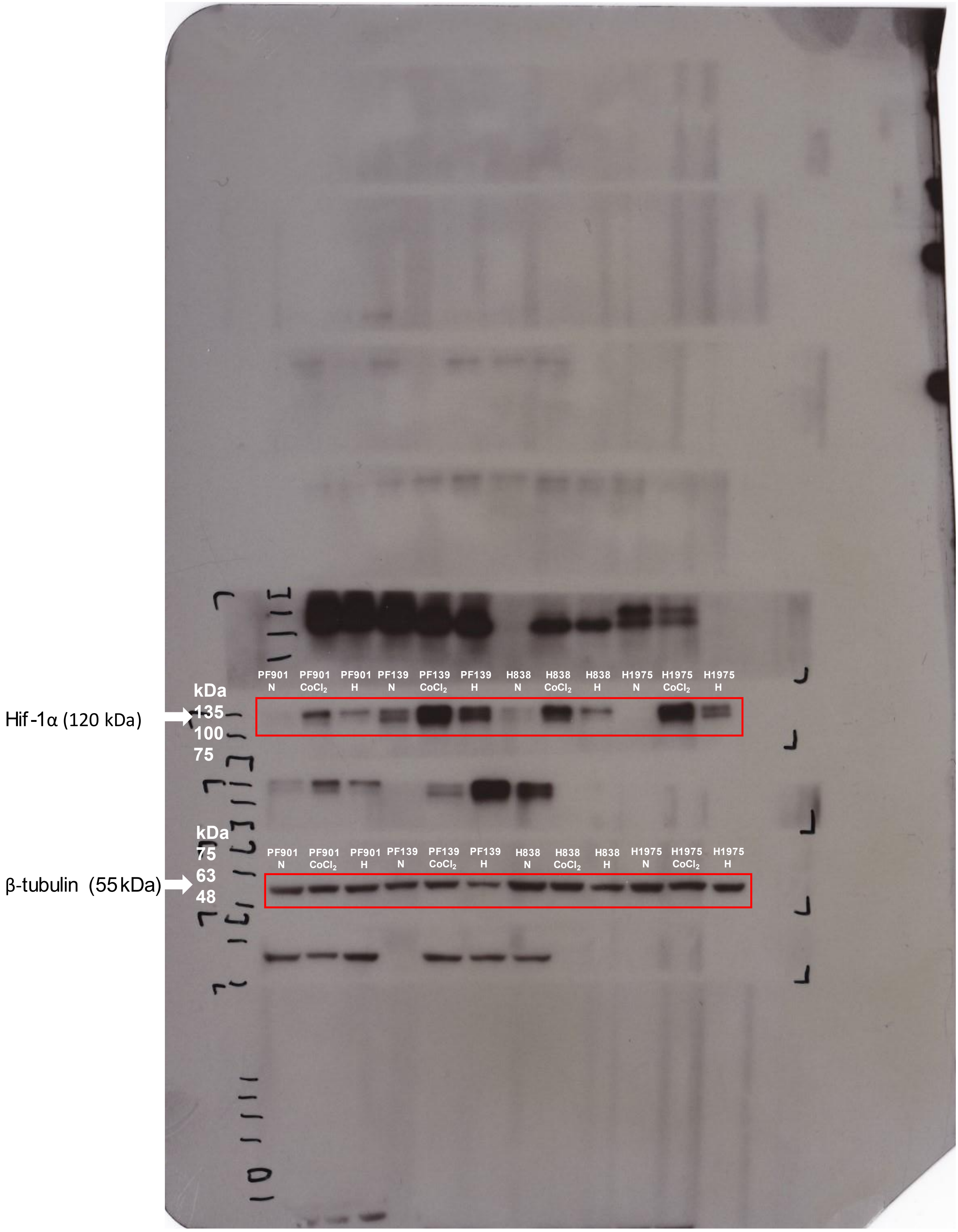

PF901 N
PF901 CoCl2
PF901 H
PF139 N
PF139 CoCl2
PF139 H
H838 N
H838 CoCl2
H838 H
H1975 N
H1975 CoCl2
H1975 H
kDa
135
100
75
Hif-1α (120 kDa)
PF901 N
PF901 CoCl2
PF901 H
PF139 N
PF139 CoCl2
PF139 H
H838 N
H838 CoCl2
H838 H
H1975 N
H1975 CoCl2
H1975 H
kDa
75
63
48
β-tubulin (55kDa)

WB for p-P38, P38, p-histoneH3, p-SRC, p-FAK, FAK,

Total protein with Protein MW marker (5-245 kDa) (Prestained ProteinSHARPMASS™ VI, EuroClone S.p.A., 6 Pero, Italy)

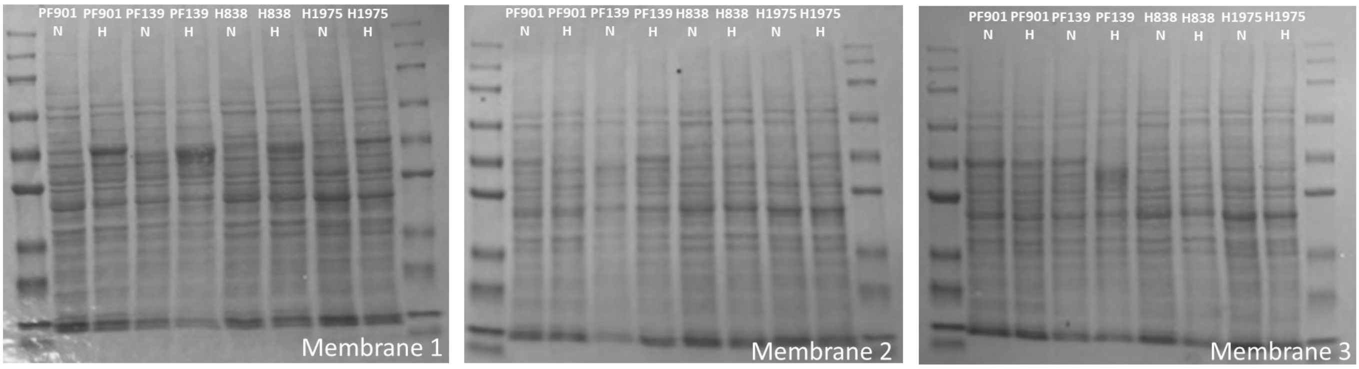

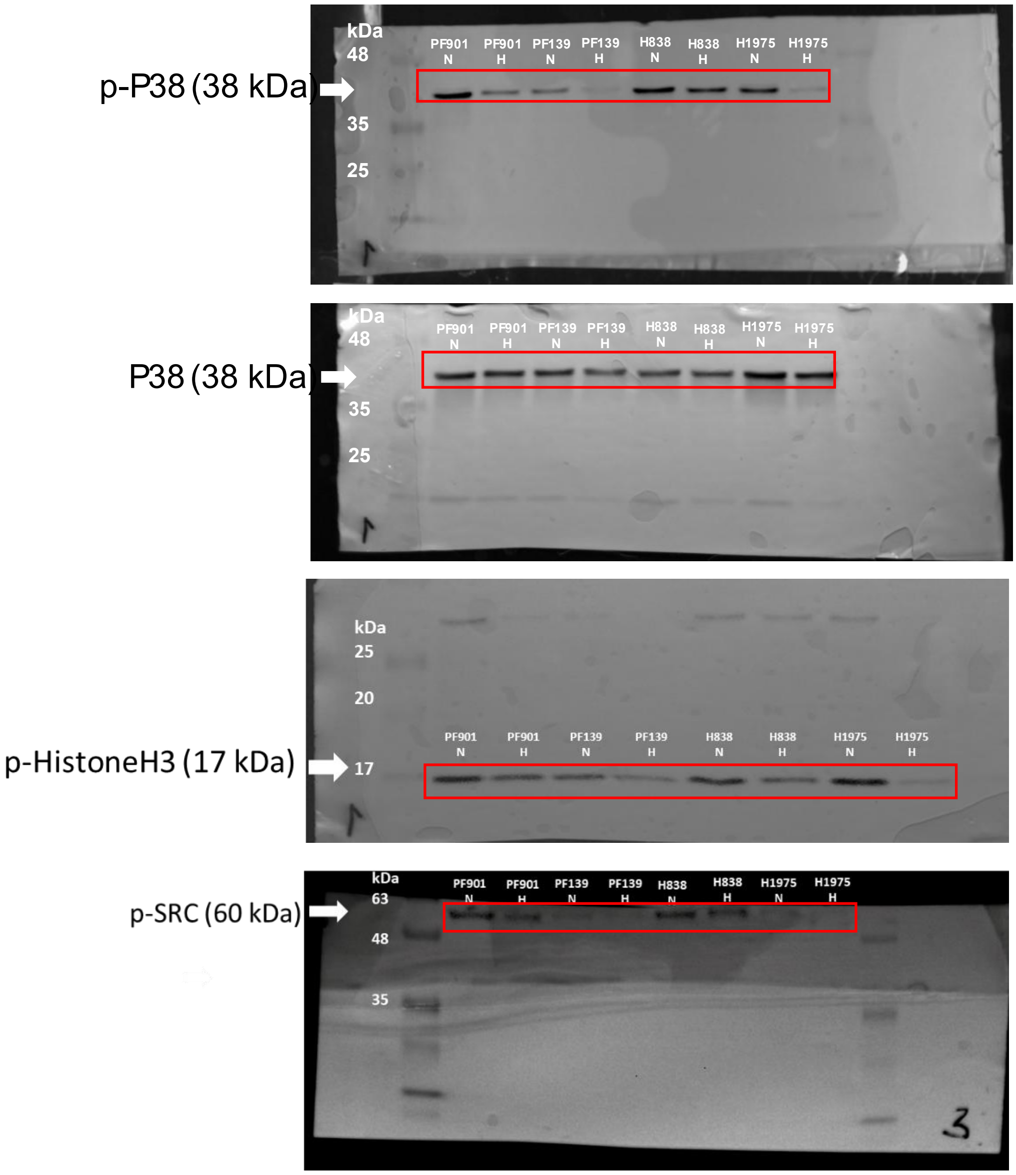

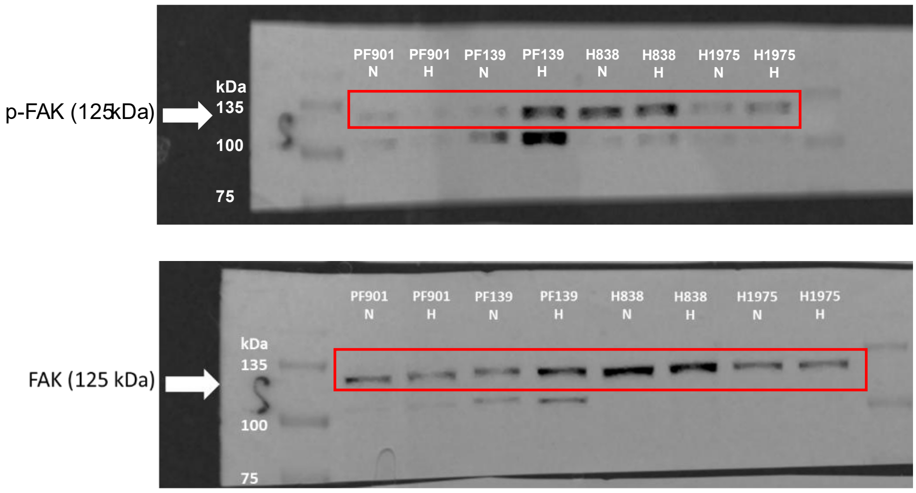
p-FAK (125kDa)
PF901 N
PF901 H
PF139 N
PF139 H
H838 N
H838 H
H1975 N
H1975 H
kDa
135
100
75
FAK (125 kDa)
PF901 N
PF901 H
PF139 N
PF139 H
H838 N
H838 H
H1975 N
H1975 H
kDa
135
100
75
