# Supplementary material for: Differential effects of hypoxia on motility using various in vitro models of lung adenocarcinoma
Source: Sci Rep. 2024 Sep 3;14:20482. doi: 10.1038/s41598-024-70769-w (PMC11372077; doi:10.1038/s41598-024-70769-w)
Supplement: Supplementary file 3 — Supplementary Video 1. [file 41598_2024_70769_MOESM3_ESM.pptx]

## Slide 1
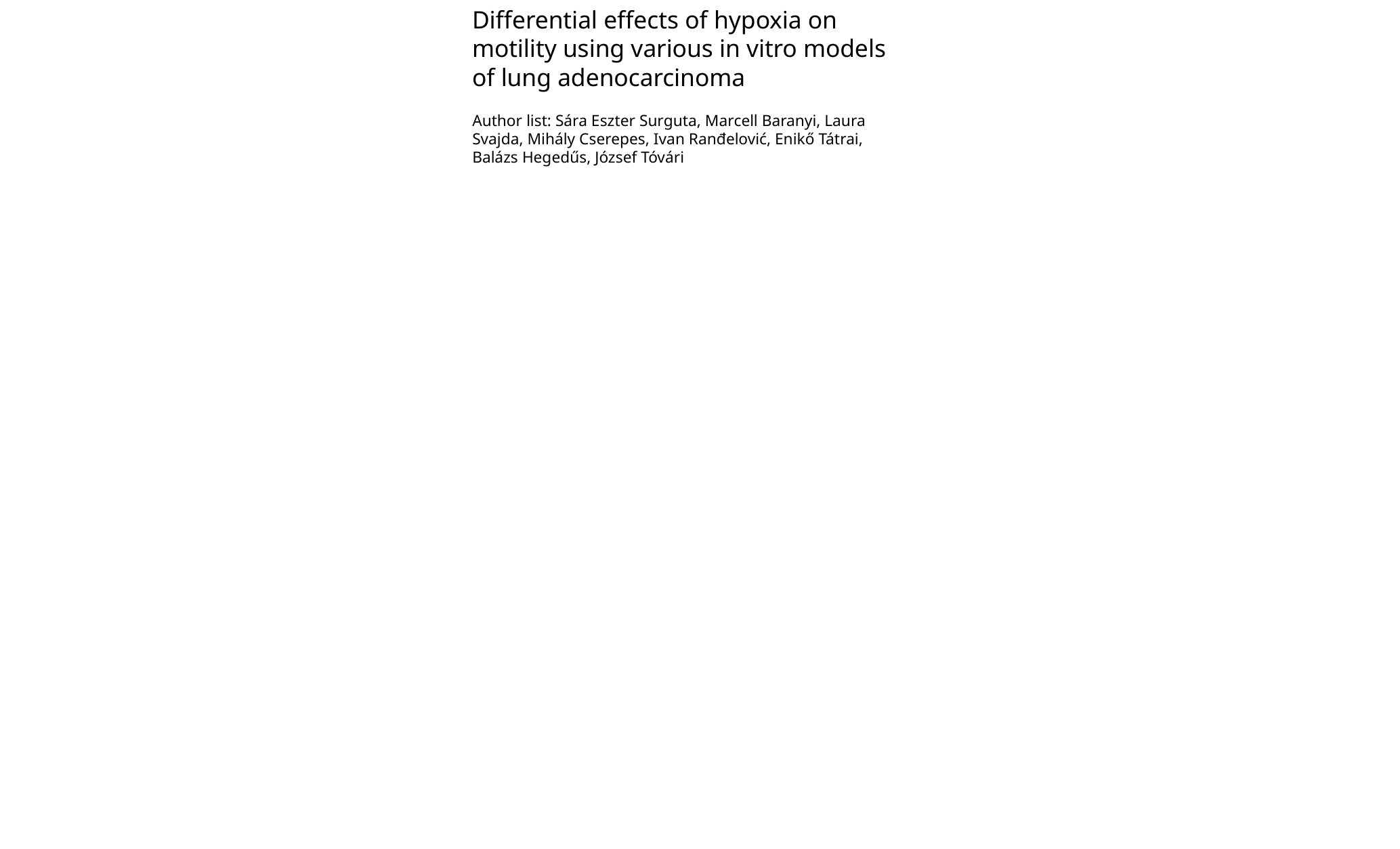

Differential effects of hypoxia on motility using various in vitro models of lung adenocarcinoma
Author list: Sára Eszter Surguta, Marcell Baranyi, Laura Svajda, Mihály Cserepes, Ivan Ranđelović, Enikő Tátrai, Balázs Hegedűs, József Tóvári

## Slide 2
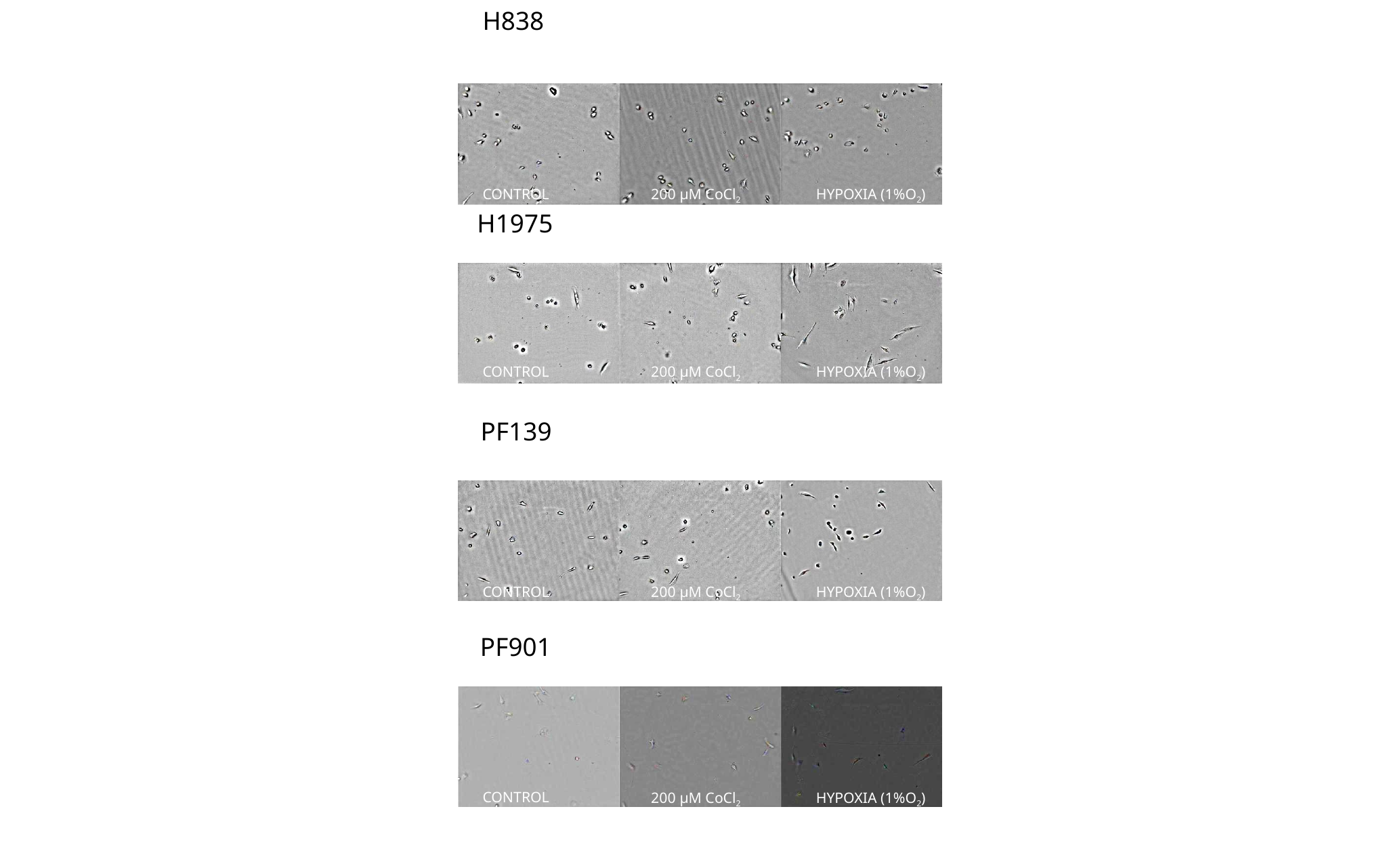

H838
CONTROL
200 μM CoCl2
HYPOXIA (1%O2)
H1975
CONTROL
200 μM CoCl2
HYPOXIA (1%O2)
PF139
CONTROL
200 μM CoCl2
HYPOXIA (1%O2)
PF901
CONTROL
200 μM CoCl2
HYPOXIA (1%O2)

## Slide 3
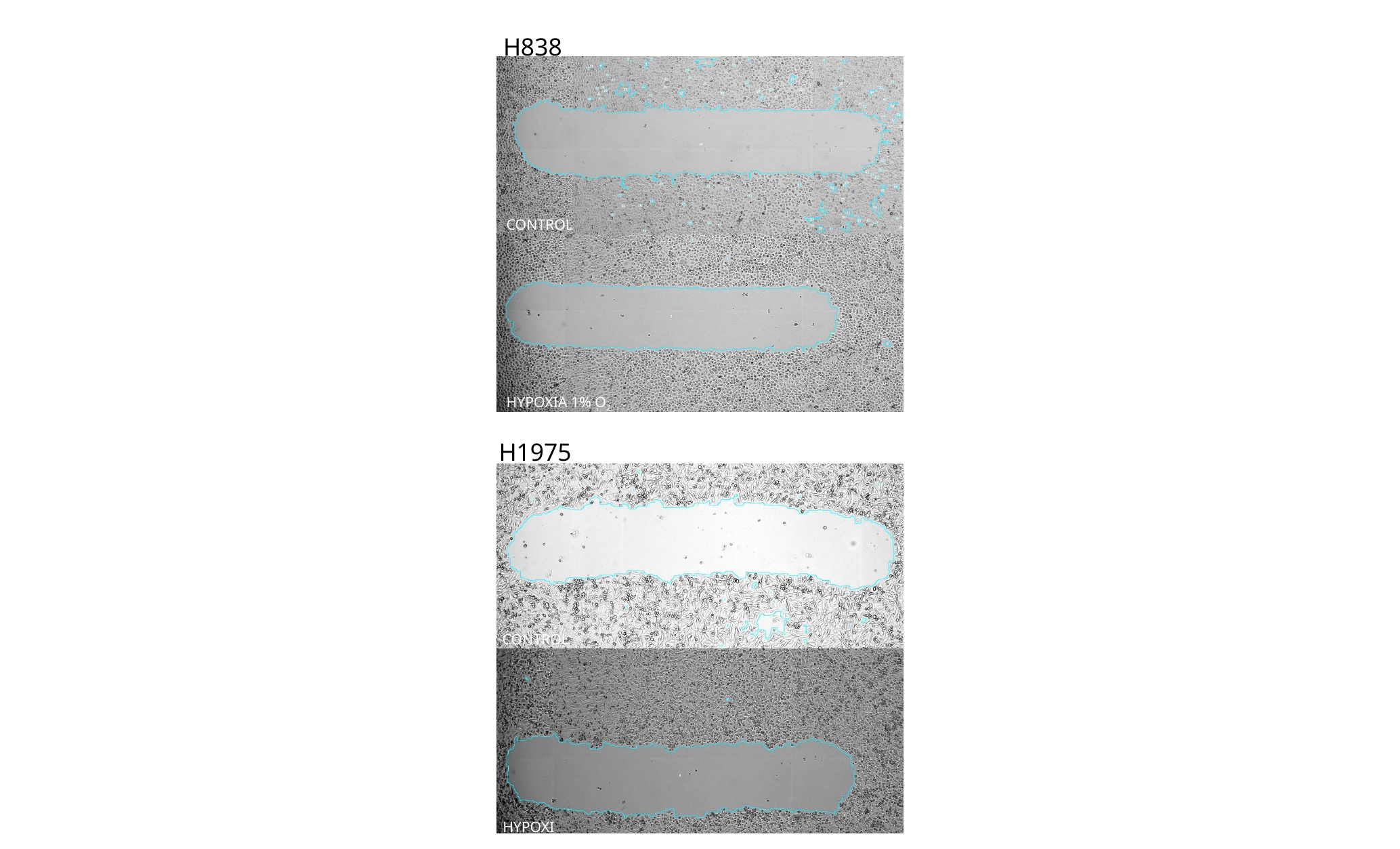

H838
CONTROL
HYPOXIA 1% O2
H1975
CONTROL
HYPOXIA
CONTROL
HYPOXIA

## Slide 4
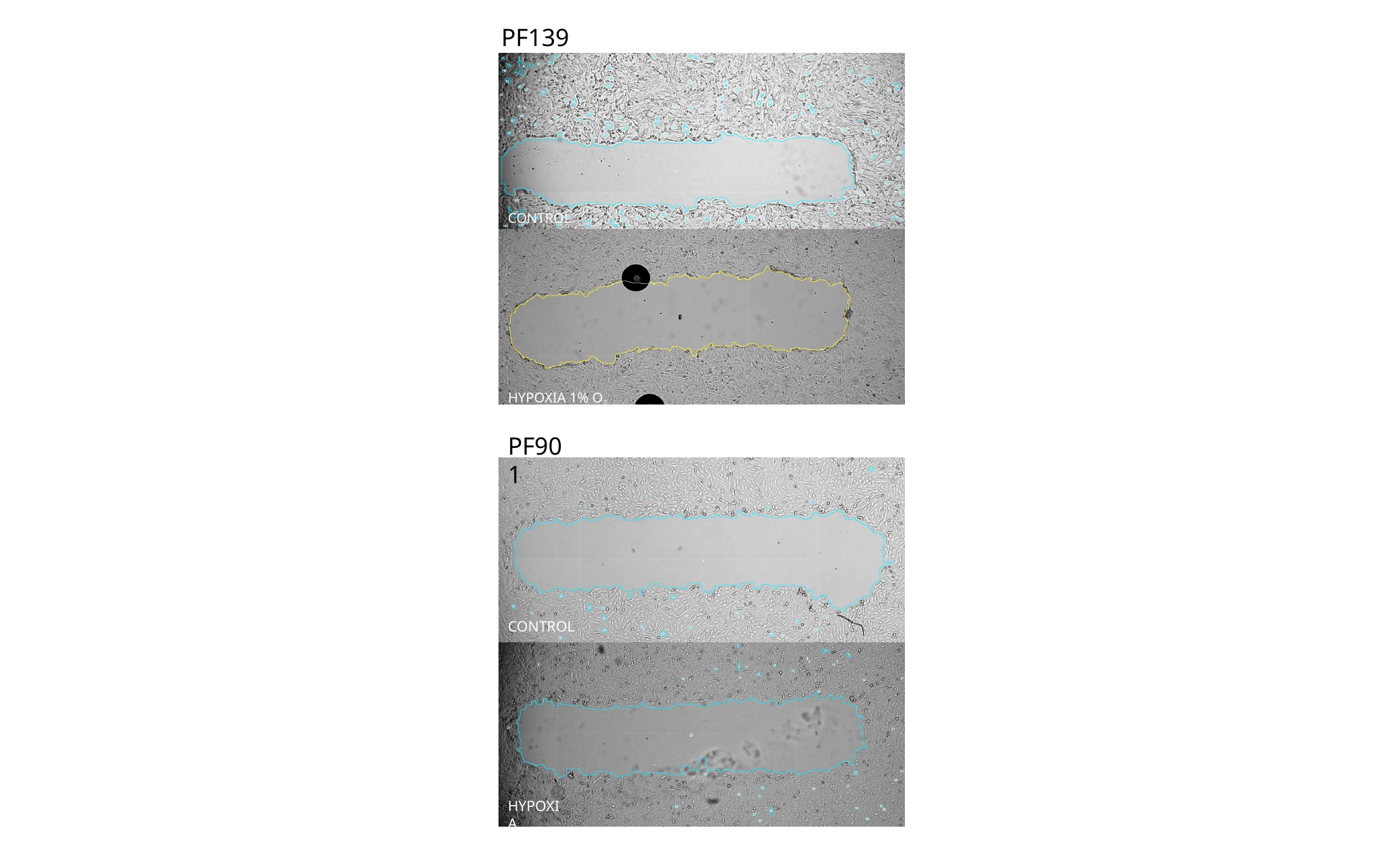

PF139
CONTROL
HYPOXIA 1% O2
PF901
CONTROL
HYPOXIA
